# Diffusion of Alkaline Metals in Two-Dimensional β_1_-ScSi_2_N_4_ and β_2_-ScSi_2_N_4_ Materials: A First-Principles Investigation

**DOI:** 10.3390/nano15161268

**Published:** 2025-08-16

**Authors:** Ying Liu, Han Fu, Wanting Han, Rui Ma, Lihua Yang, Xin Qu

**Affiliations:** Key Laboratory of Functional Materials Physics and Chemistry of the Ministry of Education, Key Laboratory of Preparation and Application of Environmental Friendly Materials, College of Physics, Jilin Normal University, Changchun 130103, China; 233120967@mails.jlnu.edu.cn (Y.L.); f13044302203@163.com (H.F.); 15567090213@163.com (W.H.); ma8391438@163.com (R.M.)

**Keywords:** MA_2_Z_4_, metal materials, two-dimensional anode materials, battery, first-principles calculations

## Abstract

The MA_2_Z_4_ family represents a class of two-dimensional materials renowned for their outstanding mechanical properties and excellent environmental stability. By means of elemental substitution, we designed two novel phases of ScSi_2_N_4_, namely β_1_ and β_2_. Their dynamical, thermal, and mechanical stabilities were thoroughly verified through phonon dispersion analysis, ab initio molecular dynamics (AIMD) simulations, and calculations of mechanical parameters such as Young’s modulus and Poisson’s ratio. Electronic structure analysis using both PBE and HSE06 methods further revealed that both the β_1_ and β_2_ phases exhibit metallic behavior, highlighting their potential for battery-related applications. Based on these outstanding properties, the climbing image nudged elastic band (CI-NEB) method was employed to investigate the diffusion behavior of Li, Na, and K ions on the material surfaces. Both structures demonstrate extremely low diffusion energy barriers (Li: 0.38 eV, Na: 0.22 eV, K: 0.12 eV), indicating rapid ion migration—especially for K—and excellent rate performance. The lowest barrier for K ions (0.12 eV) suggests the fastest diffusion kinetics, making it particularly suitable for high-power potassium-ion batteries. The significantly lower barrier for Na ions (0.22 eV) compared with Li (0.38 eV) implies that both β_1_ and β_2_ phases may be more favorable for fast-charging/discharging sodium-ion battery applications. First-principles calculations were applied to determine the open-circuit voltage (OCV) of the battery materials. The β_2_ phase exhibits a higher OCV in Li/Na systems, while the β_1_ phase shows more prominent voltage for K. The results demonstrate that both phases possess high theoretical capacities and suitable OCVs.

## 1. Introduction

Since the isolation of graphene in 2004, two-dimensional (2D) materials have attracted extensive research interest due to their unique structural, electronic, and mechanical properties [1]. Recent years have witnessed a surge in the exploration of novel 2D systems beyond conventional materials such as MoS_2_ [2], WS_2_ [3], and [4]. These materials are now widely studied for applications in electronics, optoelectronics, sensing, and energy storage. The emergence of atomically thin materials with tailored compositions has greatly enriched the 2D material family. Among them, the MA_2_Z_4_ (M = transition metal, A = group IV element, Z = N or other pnictogens/chalcogens) system represents a new frontier [5,6]. Notably, MoSi_2_N_4_ was synthesized experimentally via chemical vapor deposition (CVD) in 2020, marking the first realization of this class with high environmental stability and potential for integration in flexible electronic devices [7]. Although the majority of research to date remains theoretical, recent advances suggest that MA_2_Z_4_ materials could serve in real-world applications such as field-effect transistors, piezoelectric devices, and possibly as electrode materials in batteries [8], though experimental demonstrations remain limited.

The MA_2_Z_4_ family [9], including the archetypal MoSi_2_N_4_, has garnered increasing attention for its structural tunability via isoelectronic substitution. By systematically varying M (e.g., Mo, W, Cr, V, Sc), A (Si, Ge), and Z (N, P, As), researchers have predicted a wide range of stable configurations, each with distinct electronic, magnetic, or optoelectronic properties [10,11,12]. In particular, MSi_2_N_4_ [13,14] systems have shown promising potential, with members from early transition metals (e.g., groups III–VI) offering diverse physical behaviors due to d-orbital contributions. However, many of these studies are based solely on first-principles calculations, and caution must be exercised when extrapolating potential applications without experimental validation. Properties such as carrier mobility, band gap modulation, and magnetic ordering have been predicted, yet only a limited number of MA_2_Z_4_ structures have been synthesized and characterized to date. Among these, MoSi_2_N_4_ remains the most well-studied [15,16], underscoring the need to explore other group and period combinations experimentally to fully assess the potential of this materials platform.

Recent theoretical investigations have expanded the MA_2_Z_4_ family to include the ScSi_2_N_4_ system, a previously unexplored compound that exhibits promising characteristics for energy storage applications. To date, the known studies on ScSi_2_N_4_ include [17,18]. In this work, two structurally stable polymorphs—β_1_-ScSi_2_N_4_ and β_2_-ScSi_2_N_4_—were designed and evaluated using density functional theory (DFT). This study focused on their viability as anode materials for Li-, Na-, and K-ion batteries. The diffusion energy barriers for alkali metal ions were found to be exceptionally low: 0.38 eV (Li), 0.22 eV (Na), and 0.12 eV (K) for both β_1_ and β_2_ phases, indicating excellent ion mobility. These values are competitive with or superior to those of conventional 2D anode materials such as phosphorene or Ti_3_C_2_ MXenes [19]. The relevance of these low barriers underscores the potential of ScSi_2_N_4_-based materials in next-generation rechargeable battery technologies, especially considering their favorable open-circuit voltages and theoretical capacities. As such, the exploration of Sc-based MA_2_N_4_ systems could significantly broaden the applicability of this emerging materials family.

## 2. Computational Details

First-principles calculations were carried out within the framework of density functional theory (DFT) [20] using the Vienna Ab initio Simulation Package (VASP 5.4) [21]. The interaction between ions and electrons was treated using the projector augmented-wave (PAW) [22] method, and the exchange–correlation energy was described by the generalized gradient approximation (GGA) [23] in the form proposed by Perdew, Burke, and Ernzerhof (PBE) [24]. The HSE06 hybrid functional (Heyd–Scuseria–Ernzerhof) [25] was employed to achieve accurate bandgap predictions by mixing exact exchange with PBE exchange–correlation energy. To ensure computational accuracy, the plane-wave energy cutoff was set to 520 eV, and the energy convergence criterion was set to 10^−6^ eV. During structural optimization, all atomic positions were relaxed until the residual force on each atom was less than 10^−3^ eV/Å. A supercell with a vacuum layer of approximately 30 Å was constructed to eliminate spurious interactions between periodic images along the out-of-plane direction. For the analysis of dynamical stability, phonon dispersion relations were calculated using the Phonopy package (2.22.1) [26] based on the supercell approach. In addition, ab initio molecular dynamics (AIMD) simulations were conducted under the canonical (NVT) ensemble to evaluate thermal stability at various temperatures. Each simulation lasted for 10 ps with a time step of 2.0 fs, and temperature control was achieved using the Nosé–Hoover thermostat [27]. The elastic constants *C*_11_, *C*_22_, and *C*_66_ for the 2D β_1_-ScSi_2_N_4_ and β_2_-ScSi_2_N_4_ structures were obtained by applying a series of small strains to the fully relaxed structures using the Vienna Ab initio Simulation Package (VASP) and calculating the resulting stress tensors. The elastic constants were then extracted from the linear stress–strain relationships using the VASPKIT package (1.5.1) [28]. The diffusion behavior of Li, Na, and K ions on the material surface was investigated using the climbing image nudged elastic band (CI-NEB) method to determine the migration pathways and corresponding energy barriers, revealing their potential for energy storage applications. To calculate the open-circuit voltage (OCV) of battery materials, we adopted a thermodynamic approach, first computing the Gibbs free energy change (ΔG) during Li/Na/K intercalation in the electrode material, then deriving the voltage via the Nernst equation. For consistency, all compared data were obtained using the PBE-GGA functional unless otherwise noted. Previous studies [25] have shown that while hybrid functionals such as HSE06 can affect the predicted band gap of MA_2_Z_4_ compounds, the differences in adsorption energy and ion diffusion barriers between PBE and HSE06 remain minor. Thus, our comparisons are considered valid within the accepted computational accuracy for battery material screening. To evaluate the practical competitiveness of ScSi_2_N_4_ as an anode material, we compared its theoretical electrochemical performance with several extensively studied two-dimensional materials reported in the literature [29,30]. Although these materials differ in structure and composition, they serve as valid references for assessing electrochemical performance. To further evaluate the electrochemical performance of ScSi_2_N_4_-based electrodes, we compared our calculated results with those reported for other two-dimensional anode materials. These references were selected because they provide relevant performance benchmarks. We note that the reported values for these materials were obtained using different DFT methods (e.g., PBE, PBE + U, HSE06), which can lead to quantitative differences in properties such as adsorption energies, diffusion barriers, and voltages. Therefore, these comparisons are intended as qualitative indicators of relative performance rather than exact numerical matches. This approach highlights the competitive advantages of the ScSi_2_N_4_ phase, particularly in terms of ion mobility and capacity, while also acknowledging the differences between the different research methods. The formulas for Young’s modulus and Poisson’s ratio are as follows: $Y_{x}={[C}_{11}C_{22}-C_{12}^{2}]/C_{22}$ and $Y_{y}=\frac{\left[C_{11}C_{12}-C_{12}^{2}\right]}{C_{11}}$; $V_{x}=\frac{C_{12}}{C_{22}}$, $V_{y}=\frac{C_{12}}{C_{11}}$.

## 3. Results and Discussion

### 3.1. Structural Information and Stability of 2D β_1_-ScSi_2_N_4_ and β_2_-ScSi_2_N_4_

**Figure 1:**
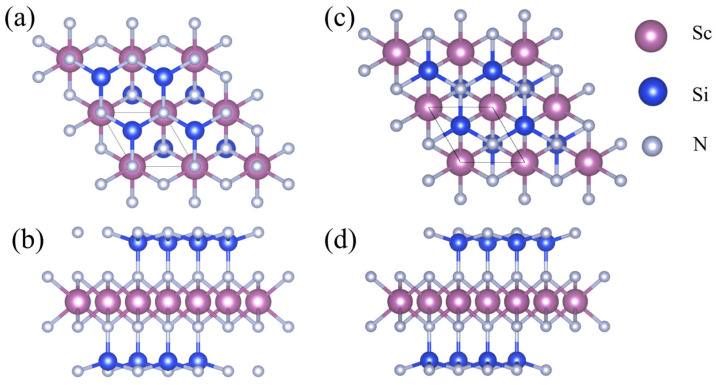
(**a**) Front view and (**b**) side view of β_1_-ScSi_2_N_4_; (**c**) front view and (**d**) side view of β_2_-ScSi_2_N_4._ Pink, blue, and gray balls represent Sc, Si, and N atoms, respectively.

Both β_1_-ScSi_2_N_4_ and β_2_-ScSi_2_N_4_ have a hexagonal lattice structure consisting of seven atomic layers, as shown in Figure 1. It can be regarded as the insertion of a layer of MoS_2_-type ScN_2_ into a layer of InSe-type Si_2_N_2_ monolayer; that is, sandwiching the ScN_2_ layer between two SiN layers to form a hexagonal ScSi_2_N_4_ monolayer structure, which belongs to the P6m1 space group. The optimized lattice constant of β_1_-ScSi_2_N_4_ is a = b = 3.00 Å, and the optimized lattice constant of β_2_-ScSi_2_N_4_ is a = b = 2.98 Å. Both unit cells contain one Sc atom, two Si atoms, and four N atoms, and each Sc atom and Si atom are surrounded by six and four adjacent N atoms, respectively. β_1_-ScSi_2_N_4_ belongs to the P-3m1 (164) space group, and β_2_-ScSi_2_N_4_ belongs to the P3m1 (156) space group.

**Figure 2:**
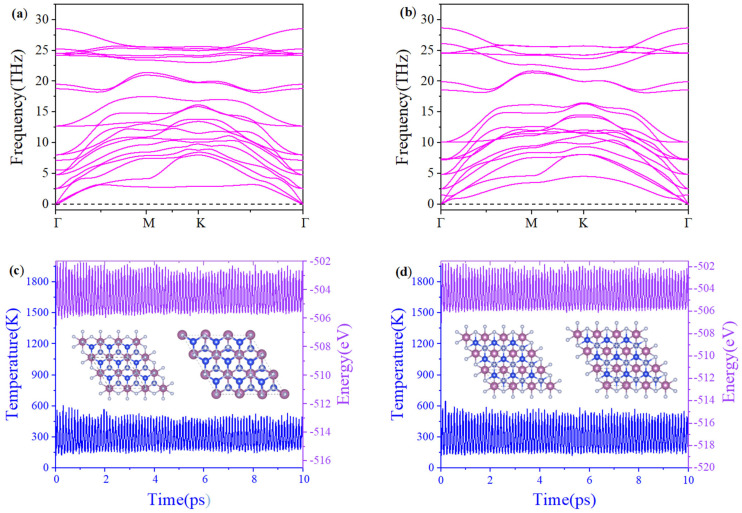
Phonon spectra of (**a**) β_1_-ScSi_2_N_4_ and (**b**) β_2_-ScSi_2_N_4_, molecular dynamics of (**c**) β_1_-ScSi_2_N_4_ and (**d**) β_2_-ScSi_2_N_4_ at 300K for 10ps. The letters “Γ”, “M”, and “K” correspond to the high-symmetry points.

The stability calculation of two-dimensional materials is the bridge between theoretical predictions and experimental applications, and directly determines whether the material can be synthesized, whether it can be used in practice, and whether it has unique physical and chemical properties. In the phonon calculation, the phonon dispersion of β_1_-ScSi_2_N_4_ and β_2_-ScSi_2_N_4_ does not exhibit any imaginary frequency, confirming their dynamic stability, as shown in Figure 2a,b. The maximum frequency of 2D β_1_-ScSi_2_N_4_ is 27.7 THz (925 cm^−1^), while 2D β_2_-ScSi_2_N_4_ is 28.6 THz (954 cm^−1^), which is slightly lower than for VSi_2_N_4_ (992 cm^−1^) [14] but sufficiently higher than MoS_2_ (473 cm^−1^) [31], showing the high stability of 2D β_1_-ScSi_2_N_4_ and β_2_-ScSi_2_N_4_. Ab initio molecular dynamics (AIMD) simulations were performed using a 3 × 3 × 1 supercell for the β_1_-ScSi_2_N_4_ and β_2_-ScSi_2_N_4_ monolayers at 300 K. Within 10 ps, there is no obvious displacement of the atomic positions in the structure, no obvious breakage of the chemical bonds, and the structure remains intact, indicating good thermal stability, as shown in Figure 2c,d. Cohesive energy is a physical quantity that describes the binding strength between atoms or molecules in a material. It reflects the material’s stability: the more negative the value, the stronger the atomic bonding and the more stable the material. The cohesive energy of β_1_-ScSi_2_N_4_ is −7.78 eV/atom. The cohesive energy of β_2_-ScSi_2_N_4_ is −7.79 eV/atom. The cohesive energies of β_1_-ScSi_2_N_4_ and β_2_-ScSi_2_N_4_ are more negative than those of YSi_2_N_4_ [32] (−4.294 eV/atom), Cr_2_BN [33] (−4.06 eV/atom), and Fe_2_Si [34] (−4.10 eV/atom). The negative cohesive energies indicate structural stability, demonstrating the experimental feasibility of synthesizing β_1_-ScSi_2_N_4_ and β_2_-ScSi_2_N_4_.

**Figure 3:**
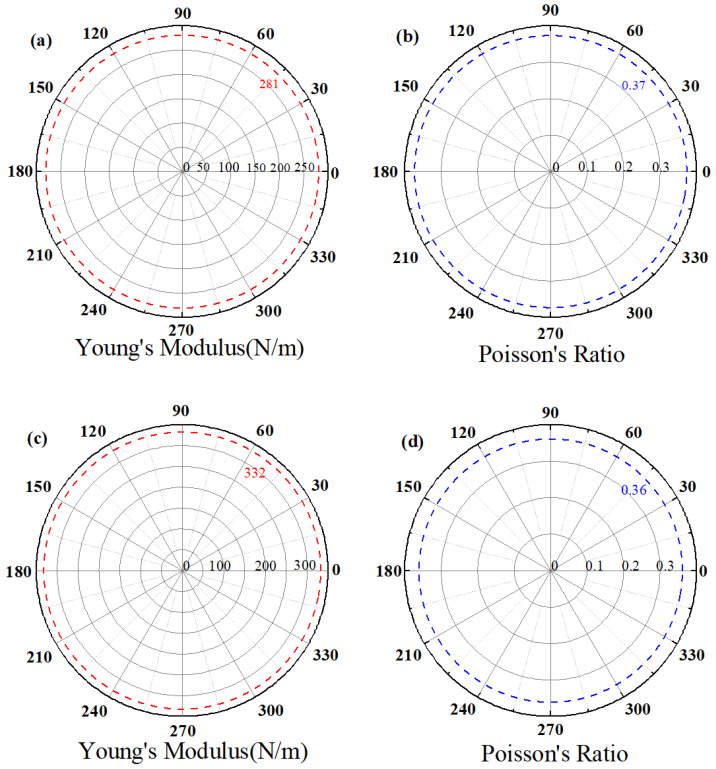
Young’s modulus and Poisson’s ratio of (**a**,**b**) β_1_-ScSi_2_N_4_ and (**c**,**d**) β_2_-ScSi_2_N_4_.

In β_1_-ScSi_2_N_4_, the elastic constants are C_11_ = 325.96 N/m, C_22_ = 121.65 N/m, and C_66_ = (C_11_ − C_12_)/2 = 102.16 N/m. For β_2_-ScSi_2_N_4_, these values are C_11_ = 381.83 N/m, C_22_ = 137.80 N/m, and C_66_ = (C_11_ − C_12_)/2 = 122.01 N/m. These elastic constants satisfy the Born criteria for hexagonal systems [35,36] (C_11_ > 0 and C_11_ > |C_12_|), confirming their mechanical stability. Figure 3 demonstrates that the angle-dependent Young’s modulus and Poisson’s ratio exhibit remarkable isotropy, indicating consistent mechanical properties in all directions, which facilitates practical applications of these materials. The magnitude of Young’s modulus reflects material stiffness—higher values correspond to greater resistance to deformation. The β_1_-ScSi_2_N_4_ monolayer shows a Young’s modulus of 281 N m^−1^, while β_2_-ScSi_2_N_4_ exhibits a higher value of 332 N m^−1^. These values surpass those of Ti_2_C (130 N m^−1^) [37] and MoS_2_ (119 N m^−1^) [38]. The Poisson’s ratio, describing the transverse strain response to longitudinal stress, is 0.37 for β_1_-ScSi_2_N_4_ and 0.36 for β_2_-ScSi_2_N_4_, comparable to CrAsS_4_ (0.34) [39] and MoS_2_ (0.31) monolayers. These results confirm that both β_1_-ScSi_2_N_4_ and β_2_-ScSi_2_N_4_ possess excellent tensile/compressive resistance within their elastic limits.

### 3.2. Electronic Properties of 2D β_1_-ScSi_2_N_4_ and β_2_-ScSi_2_N_4_

**Figure 4:**
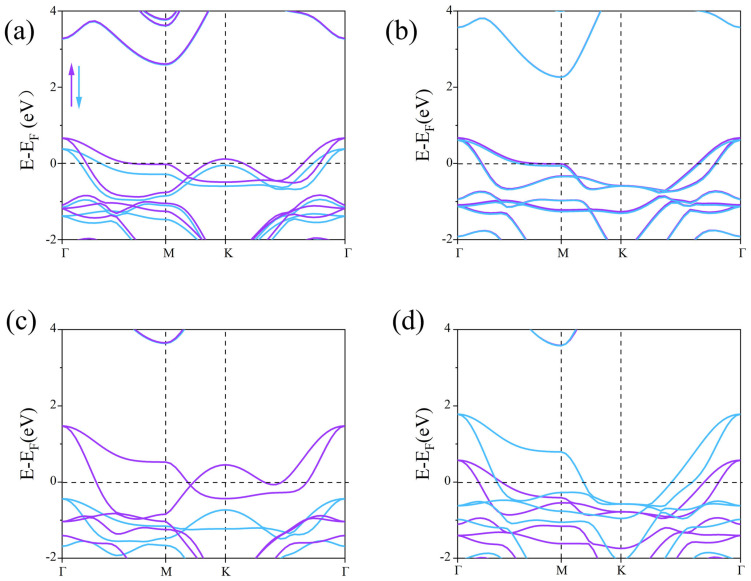
Electronic band structure of ((**a**,**b**), the upside line) β_1_-ScSi2N4 and β_2_-ScSi2N4 calculated from GGA + PBE methods; ((**c**,**d**), the downside line) β_1_-ScSi_2_N_4_ and β_2_-ScSi_2_N_4_ calculated from HSE06 methods. Purple represents the spin-up energy band, blue represents the spin-down energy band, and the black dotted line marks the Fermi level position.

Next, the spin-polarized band structures were calculated using the PBE and HSE06 methods to explore the electronic properties of the β_1_-ScSi_2_N_4_ and β_2_-ScSi_2_N_4_ monolayers (Figure 4). In the PBE calculations, we found that the spin-up and spin-down channels of β_1_-ScSi_2_N_4_ and β_2_-ScSi_2_N_4_ cross the Fermi level, indicating their intrinsic metallic nature. However, the PBE functional typically underestimates band gaps. Therefore, we further calculated the band structures of the β_1_-ScSi_2_N_4_ and β_2_-ScSi_2_N_4_ monolayers using the HSE06 method.

**Figure 5:**
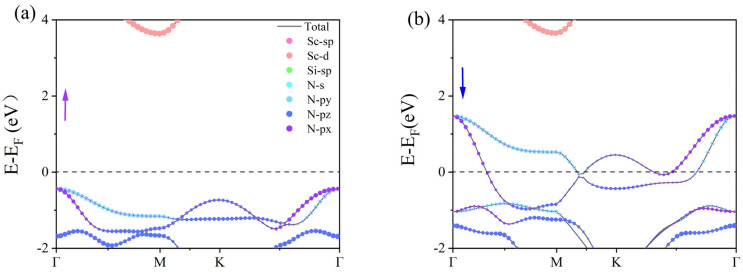
Orbital projection band structure and spin projection density of β_2_-ScSi_2_N_4_ ((**a**) represents spin-up, while (**b**) represents spin-down). Purple represents the spin-up energy band, blue represents the spin-down energy band, and the black dotted line marks the Fermi level position.

With the inclusion of HSE06 (Figure 4c,d) we observed that the spin-down band of β_1_-ScSi_2_N_4_ crosses the Fermi level, exhibiting metallic behavior, while the spin-up band lies below the Fermi level. The valence band at the Γ point is primarily contributed by N atoms, whereas the conduction band at the M point is mainly dominated by Sc atoms. A band gap of approximately 4.0 eV exists between the conduction and valence bands, resulting in an overall half-metallic character. The presence of half-metallicity implies vacant states in the band structure, leading to spin asymmetry and partially filled valence electron arrangements. Due to the significantly higher electronegativity of N compared with Sc and Si, its ability to attract electrons is much stronger. Based on the Bader charge [40] analysis shown in Table S1, we calculated the charge distribution of Sc, Si, and N atoms. The results show that N atoms are negatively charged, indicating their strong electron-withdrawing ability, while Sc and Si atoms are positively charged, indicating that electrons are mainly transferred to N atoms, supporting their strong electronegativity. Additionally, through orbital-projected band structure analysis, we demonstrated that the half-metallic nature primarily originates from the N atoms, as illustrated in Figure 5.

**Figure 6:**
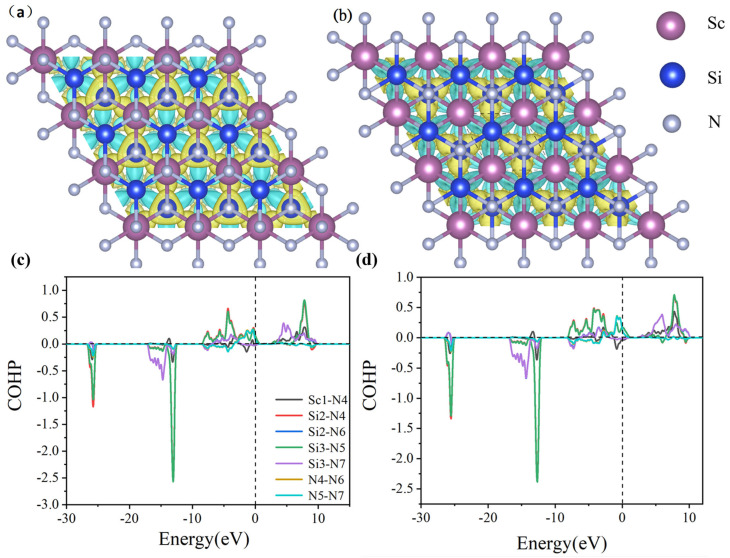
Difference charge density of (**a**) β_1_-ScSi_2_N_4_ and (**b**) β_2_-ScSi_2_N_4._ Blue represents electron loss, while yellow indicates electron gain; COHP diagrams of (**c**) β_1_-ScSi_2_N_4_ and (**d**) β_2_-ScSi_2_N_4_. Pink, blue, and gray balls represent Sc, Si, and N atoms, respectively.

PBE notoriously underestimates bandgaps, while HSE06 shows excellent agreement with experimental values, justifying its use for electronic structure analysis. Consequently, β_1_-ScSi_2_N_4_ holds promising potential for applications in magnetism, optics, and related fields. In the HSE06 calculations, the spin-up and spin-down bands of β_2_-ScSi_2_N_4_ both cross the Fermi level, maintaining their metallic nature. However, while the spin-up and spin-down states were initially degenerate, the inclusion of HSE06 leads to band splitting, with the spin-down valence band shifting to higher energies. Based on the HSE06 results, β_1_-ScSi_2_N_4_ exhibits a half-metallic character, whereas β_2_-ScSi_2_N_4_ remains metallic, thereby expanding the potential applications of the MA_2_Z_4_ family. COHP (crystal orbital Hamilton population) analysis helps to distinguish bonding, anti-bonding, and non-bonding interactions by evaluating the energy-resolved overlap of electronic states between atom pairs. The ionic bonding character is confirmed as the near-zero ICOHP values (≈0) observed in the COHP curves near the Fermi level (−2 to 2 eV) indicate negligible electronic state overlap in the Sc-N/Si-N bonding (Figure 6c,d). Furthermore, differential charge density and crystal orbital Hamiltonian population (COHP) analyses confirm that the Sc-N and Si-N bonds in both β_1_-ScSi_2_N_4_ and β_2_-ScSi_2_N_4_ exhibit ionic characteristics, as illustrated in Figure 6.

### 3.3. β_1_-ScSi_2_N_4_ and β_2_-ScSi_2_N_4_ Performance as Anode Materials

The evaluation of binding energies for Li/Na/K atoms on substrate materials is crucial for assessing the performance of ion batteries. In this study, to investigate the surface adsorption behavior on β_1_-ScSi_2_N_4_, we selected four typical adsorption sites on the outermost AZ layer: the top site of the N atom (S1), the top site of the Si atom (S2), the bridge midpoint between N and Si atoms (S3), and the center site of the honeycomb ring (S4). Similarly, for the surface adsorption study on β_2_-ScSi_2_N_4_, four characteristic sites on the outermost AZ layer the adsorption points are: the top site of the Sc atom (S1), the top site of the Si atom (S2), the bridge midpoint between Sc and Si atoms (S3), and the center site of the honeycomb ring (S4) (as shown in Figure S1).

The binding energy can be calculated using the following formula:

(1) $$∆E_{b}=E_{tot}\left(Sc{Si}_{2}N_{4}\right)+E_{tot}\left(M\right)-E_{tot}\left(MSc{Si}_{2}N_{4}\right),\left(M=Li,Na,K\right)$$

In the binding energy calculations, $E_{tot}\left(Sc{Si}_{2}N_{4}\right)$ represents the total energy of the pristine structure without adsorbed atoms, $E_{tot}\left(MSc{Si}_{2}N_{4}\right)$ denotes the total energy of the system after Li/Na/K atom adsorption, and $E_{tot}\left(M\right)$ corresponds to the total energy of an isolated Li/Na/K atom (show in Table S2). The research results demonstrate that for the β_1_-ScSi_2_N_4_ material, Li/Na/K atoms initially positioned at the S3 site spontaneously migrate to the S2 site. In contrast, for the β_2_-ScSi_2_N_4_ material, Li/Na/K atoms at the S3 site relax to the S1 site. This indicates that the S1, S2, and S4 sites can serve as effective adsorption sites for Li/Na/K atoms in both materials. The binding energy data presented in Table S1 show that a larger $∆E_{b}$ value corresponds to a stronger interaction between the ScSi_2_N_4_ material and Li/Na/K atoms (take Li as an example). Consequently, in the β_1_-ScSi_2_N_4_ material, the S4 site exhibits the most stable adsorption for Li/Na/K atoms, while in the β_2_-ScSi_2_N_4_ material, the S1 site demonstrates optimal adsorption stability.

To gain deeper insights into the adsorption mechanism of Li, Na, and K atoms on the surface of MA_2_Z_4_ monolayer materials, a quantitative evaluation of the charge transfer between the metal atoms and the two-dimensional ScSi_2_N_4_ was carried out based on Bader charge analysis. The results indicate that Li acts as a typical electron donor during adsorption, transferring approximately 0.85 e and 0.9 e of charge to the β_1_-ScSi_2_N_4_ and β_2_-ScSi_2_N_4_ monolayers, respectively. For Na and K atoms, the amount of charge transferred to the β_1_ configuration is approximately 0.88 e and 0.84 e, respectively, and the same values are observed for the β_2_ configuration. These substantial charge transfers suggest that the adsorption of Li/Na/K atoms on the MA_2_Z_4_ surface is primarily governed by electron transfer from the metal atoms to the substrate, effectively filling the lowest unoccupied molecular orbitals (LUMO) of the ScSi_2_N_4_ monolayer. This process enhances both the adsorption stability and interfacial coupling strength.

To further elucidate the spatial distribution of the electronic structure during adsorption, the differential charge density (Δρ) was calculated for Li, Na, and K atoms adsorbed at the S4 site of β_1_-ScSi_2_N_4_ and the S1 site of β_2_-ScSi_2_N_4_. The results reveal evident regions of charge accumulation and depletion in the vicinity of the adsorption sites, indicating strong electronic coupling and electrostatic interactions between the adsorbed metal atoms and the ScSi_2_N_4_ substrate. These differential charge density maps not only corroborate the dominant role of electron transfer in the adsorption mechanism but also provide critical theoretical insight into the interfacial charge behavior of MA_2_Z_4_-based two-dimensional materials for applications in energy storage and related electrochemical systems.

(2) $$\Delta \rho =\rho \left(MSc{Si}_{2}N_{4}\right)-\rho \left(Sc{Si}_{2}N_{4}\right)-\rho \left(M\right),M=Li,Na,K$$

**Figure 7:**
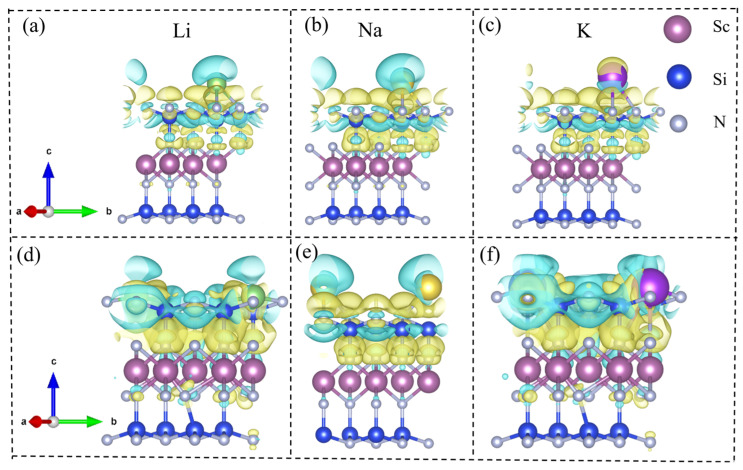
Charge density difference (CDD) of (**a**) Li, (**b**) Na, and (**c**) K atoms adsorbed at the S2 site of 2D β_1_-ScSi_2_N_4_, and (**d**) Li, (**e**) Na, and (**f**) K atoms adsorbed at the S1 site of 2D β_2_-ScSi_2_N_4_. Figures (**a**–**c**) correspond to β_1_-ScSi_2_N_4_, and figures (**d**–**f**) correspond to β_2_-ScSi_2_N_4_. Units: e/Å^3^ electrons per cubic angstrom. Regions of electron accumulation and depletion are shown in light yellow and blue, respectively. Pink, blue, and gray balls represent Sc, Si, and N atoms, respectively, while green, yellow, and purple balls represent Li, Na, and K atoms, respectively.

Here, $\rho (MSc{Si}_{2}N_{4})$ and $\rho (Sc{Si}_{2}N_{4}$) represent the electronic charge densities of the two-dimensional ScSi_2_N_4_ system with and without the adsorbed Li, Na, or K atoms, respectively. At the same time, ρ(M) denotes the charge density of an isolated Li, Na, or K atom. In the differential charge density plots shown in Figure 7, the yellow and blue regions correspond to electron accumulation and depletion, respectively. A clear charge transfer from the Li, Na, and K atoms to the ScSi_2_N_4_ monolayer is observed, indicating significant electron redistribution upon adsorption. This charge transfer leads to the formation of stable chemical bonds (M–Sc) between the adsorbed metal atoms and the substrate. Such bonding interactions are beneficial for suppressing the aggregation of metal atoms into clusters, thereby enhancing the stability of single-atom dispersion and offering theoretical support for their application in energy storage and related fields.

To determine the maximum monolayer adsorption capacity of lithium and sodium atoms on two-dimensional ScSi_2_N_4_, structural optimizations were performed using a 2 × 2 × 1 supercell (as shown in Figure S1). Li and Na ions were incrementally added in pairs to the energetically favorable adsorption sites on the ScSi_2_N_4_ surface, followed by full geometric relaxation of each configuration. The final stable structures correspond to the fully adsorbed states of Li and Na. The results indicate that up to four Li or Na atoms can be stably adsorbed on each side (top and bottom) of both β_1_-ScSi_2_N_4_ and β_2_-ScSi_2_N_4_, resulting in a total of eight Li or Na atoms per supercell. This corresponds to a maximum stoichiometry of ScSi_2_N_4_-Li_2_ and ScSi_2_N_4_-Na_2_.

Similarly, the maximum adsorption capacity for potassium atoms was found to be two atoms per side, yielding a total of four K atoms per 2 × 2 × 1 supercell. This corresponds to a stoichiometry of ScSi_2_N_4_-K_1_._5_. These findings provide important theoretical insights into the alkali metal storage capability of ScSi_2_N_4_ monolayers, which is crucial for evaluating their potential as electrode materials in alkali-ion batteries.

The calculation formula for the sequential binding energy is as follows:

(3) $$E_{sb}\left(x\right)=\frac{\left[E\left(Sc{Si}_{2}N_{4}\right)+xE\left(M\right)-E\left(M_{x}Sc{Si}_{2}N_{4}\right)\right]}{x},\left(M=Li,Na,K\right)$$

Here, $E\left(M_{x}Sc{Si}_{2}N_{4}\right)$, $E\left(Sc{Si}_{2}N_{4}\right)$, and $E\left(M\right)$ represent the total energies of the ScSi_2_N_4_ system with *x* adsorbed Li, Na, or K atoms, the pristine ScSi_2_N_4_ monolayer, and a single isolated Li, Na, or K atom, respectively. A decreasing trend in the sequential binding energy indicates that the adsorption becomes progressively less stable with increasing metal ion concentration. This can be attributed to the enhanced electrostatic repulsion between neighboring Li, Na, or K ions at higher coverages. The calculated sequential binding energies ($E_{sb}$) for Li and Na adsorption on both β_1_- and β_2_-ScSi_2_N_4_ phases remain positive across the full range of x x values (1–8), confirming that these phases can stably host up to eight Li or Na atoms without reaching adsorption saturation. In contrast, potassium adsorption shows a marked drop in binding energy at x = 8 (0.18 eV for β_1_ and 0.38 eV for β_2_), indicating that K approaches its saturation limit at high coverage and is less favorably accommodated than Li or Na. (show in Table S3) Overall, Li and Na exhibit strong and stable interactions with both ScSi_2_N_4_ polymorphs, whereas K shows limited adsorption capacity at high loading. At the same time, these results suggest that, throughout the entire adsorption process, Li, Na, and K atoms energetically prefer to bind to the 2D ScSi_2_N_4_ surface rather than aggregating into bulk metallic clusters, highlighting their favorable dispersion and potential for energy storage applications.

According to the maximum number of Li, Na, and K atoms stably adsorbed on 2D β_1_-ScSi_2_N_4_ and β_2_-ScSi_2_N_4_, the capacity is defined as follows:

(4) $$C=\frac{cF}{M\left(Sc{Si}_{2}N_{4}\right)}$$

where c represents the maximum number of intercalated ions, F is Faraday’s constant (26,801 mAh/mol), and M denotes the molecular weight. By first principles calculation we calculated the theoretical storage capacities of the β_1_ and β_2_ phases of ScSi_2_N_4_. For lithium and sodium intercalation with Li (X = 8), the capacity reaches 343.45 mA h/g, while for potassium with K, it is 171.12 mA h/g. The calculated theoretical capacities surpass those of several typical anode materials, such as TiS_2_ (240 mA h g^−1^ for Li) [41], Ta_2_CO_2_ (132.1 mA h g^−1^ for Li) [42], and YS_2_ (350 mA h g^−1^ for Na) [43]. These values are comparable to those of conventional anode materials, with the lithium and sodium capacities exhibiting excellent application potential.

The open-circuit voltage was also calculated to estimate the voltage output. The open-circuit voltage formula is:

(5) $$V=-\frac{\left[E\left(x_{2}\right)-E\left(x_{1}\right)-\left(x_{2}-x_{1}\right)E\left(M\right)\right]}{e\left(x_{2}-x_{1}\right)},\left(M=Li,Na,K\right)$$

**Figure 8:**
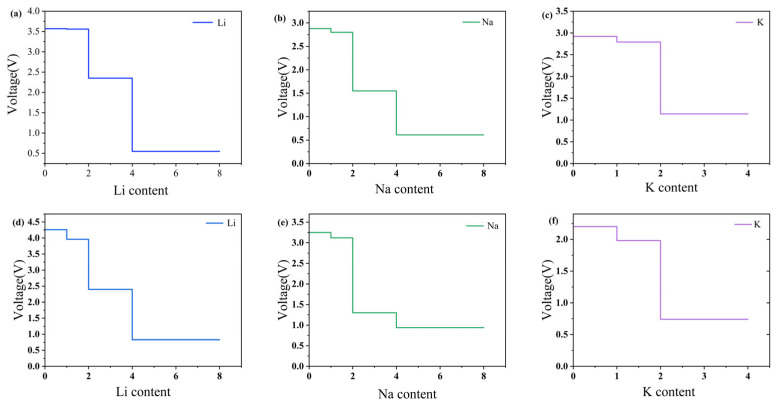
Open-circuit voltage (OCV) profiles for (**a**) Li, (**b**) Na, and (**c**) K atoms adsorbed at the S4 site of 2D β_1_-ScSi_2_N_4_, and (**d**) Li, (**e**) Na, and (**f**) K atoms adsorbed at the S1 site of 2D β_2_-ScSi_2_N_4_. Figure (**a**–**c**) correspond to β_1_-ScSi_2_N_4_, and figure (**d**–**f**) correspond to β_2_-ScSi_2_N_4_.

Here, $E\left(x_{2}\right)$, $E\left(x_{1}\right)$, and $E\left(M\right)$ denote the total energies of $Mx_{2}Sc{Si}_{2}N_{4}$, $Mx_{1}Sc{Si}_{2}N_{4}$ and a single Li, Na, or K atom, respectively. The calculated open-circuit voltages (OCVs) for both β_1_- and β_2_-ScSi_2_N_4_ exhibit a monotonic decrease with increasing alkali-metal concentration for all three ions (Li, Na, K), as illustrated in Figure 8. Throughout the entire insertion range, all OCV values remain positive, indicating thermodynamically favorable adsorption without spontaneous metal release. Notably, the final OCVs at the maximum insertion concentration are still within the practical voltage window for anode applications. These results collectively suggest that both β_1_- and β_2_-ScSi_2_N_4_ possess the key electrochemical characteristics required for high-performance Li-, Na-, and K-ion battery anodes.

**Figure 9:**
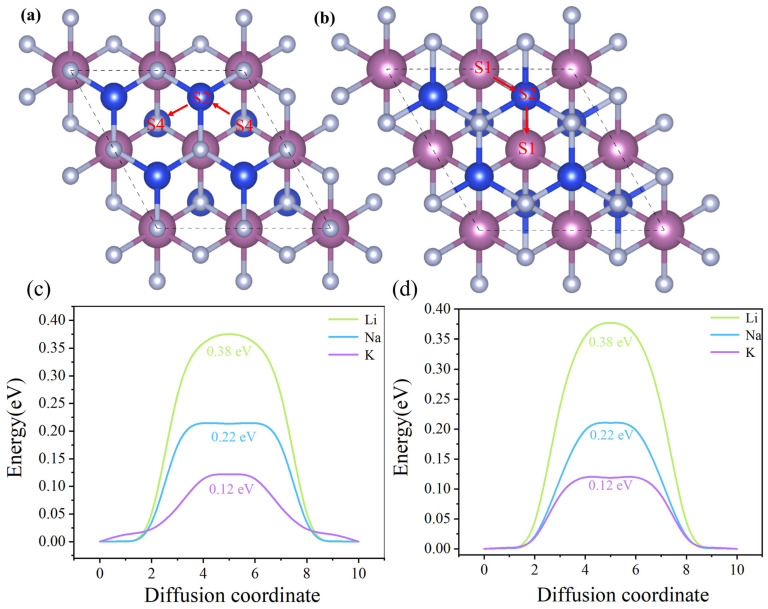
(**a**,**b**) Top view of the diffusion paths of metal ions (Li, Na, and K) on monolayers of β_1_-ScSi_2_N_4_ and β_2_-ScSi_2_N_4_, S1, S2, and S4 denote different adsorption sites, the locations along the red arrows represent the migration paths, the dashed box represents the 2 × 2 × 1 supercell used for adsorption capacity calculation. and (**c**,**d**) diffusion energy barrier profiles of Li, Na, and K ions on β_1_-ScSi_2_N_4_ and β_2_-ScSi_2_N_4_ monolayers.

Another critical feature of high-performance anode materials for rechargeable batteries is their rate capability, which originates from the mobility of the intercalated ions. To further evaluate the charge/discharge performance of ion batteries, the diffusion behavior of ScSi_2_N_4_ was investigated using the climbing image nudged elastic band (CI-NEB) method. S4 and S1 sites were chosen for simulation based on the adsorption energy analysis. One of the most favorable diffusion pathways at this site is illustrated in Figure 9a,b. The migration path of β_1_-ScSi_2_N_4_ follows S4 → S2 → S4, while that of β_2_-ScSi_2_N_4_ follows S1 → S2 → S1. The calculated diffusion energy barriers for Li, Na, and K ions in both β_1_- and β_2_-ScSi_2_N_4_ are notably low (Figure 9c,d), indicating favorable ion mobility. For comparison, these diffusion barriers are lower than or comparable to several reported anode materials, such as GeP_3_ (0.50 eV) [44] and T-graphene (0.44 eV) [29]. The diffusion barriers for Na and K are comparable to those of MoS_2_ (0.28 eV) [30] and Ti_3_C (0.18 eV) [45], respectively.

These results suggest that the 2D ScSi_2_N_4_ electrode offers excellent rate performance for rechargeable Li-, Na-, and K-ion batteries. In particular, Na ions exhibit faster diffusion than Li ions due to their larger atomic radius, which leads to a greater distance from the substrate and reduced repulsive interactions. Similarly, K ions diffuse even more rapidly than Na ions for the same reason.

To further clarify the influence of structural anisotropy on ion migration, three possible diffusion pathways were evaluated, and the lowest-barrier Path 1 was chosen as the reference. The migration energy barriers of Li^+^, Na^+^, and K^+^ along Path 2 and Path 3 in both configurations (see Figure S2 and Table S4) reveal that in β_1_, the barriers differ significantly between directions, demonstrating distinct anisotropic behavior—Li^+^ migrates more easily along Path 2, while Na^+^ and K^+^ prefer Path 3. This directional preference originates from variations in channel width and local potential fields. In contrast, β_2_ exhibits overall higher migration barriers and reduced directional differences, approaching isotropic diffusion. These findings demonstrate that the inherently low migration barriers of ScSi_2_N_4_ not only ensure excellent ion transport kinetics, but also highlight that the asymmetric channels in β_1_ can further enhance diffusion along specific directions, while the high-symmetry β_2_ suppresses such directional selectivity.

## 4. Conclusions

This study systematically investigates the structural stability, electronic properties, and potential applications of two-dimensional β_1_-ScSi_2_N_4_ and β_2_-ScSi_2_N_4_ as anode materials for rechargeable Li-, Na-, and K-ion batteries. Our first-principles calculations demonstrate that both phases possess low diffusion energy barriers for the considered alkali ions, indicating excellent ion mobility and suggesting potential for fast charge/discharge cycles. The materials also exhibit high theoretical capacities, reflecting their ability to accommodate a significant number of ions without structural degradation. Moreover, the predicted average open-circuit voltages lie within suitable ranges that align well with typical metal-ion battery operating voltages, indicating good compatibility with existing battery technologies. Taken together, these favorable structural and electrochemical properties highlight two-dimensional ScSi_2_N_4_ as a promising candidate for next-generation anode materials in diverse alkali-ion battery systems.

## Data Availability

The data that support the findings of this study are available from the corresponding author upon reasonable request.

## Supplementary Materials

The following supporting information can be downloaded at: https://www.mdpi.com/article/10.3390/nano15161268/s1, Figure S1: Schematic diagram of the four favorable adsorption sites on (a) β_1_-ScSi_2_N_4_ and (b) β_2_-ScSi_2_N_4_. In β_1_-ScSi_2_N_4_, S1, S2, S3, and S4 correspond to the top N atom above the Sc atom, the top of Si, the top of the bridge site between Si and N, and the N atom above the Si atom, respectively. In β_2_-ScSi_2_N_4_, S1, S2, S3, and S4 correspond to the top of Sc, the top of Si, the top of the bridge site between Si and Sc, and the N atom above the Si atom, respectively; Figure S2: (a–c) Maximum adsorption configurations of Li, Na, and K atoms on the β_1_-ScSi_2_N_4_ monolayer, and (d–f) on the β_2_-ScSi_2_N_4_ monolayer, in the order of Li, Na, and K; Figure S3: Ion diffusion paths and corresponding migration barrier curves for β_1_ and β_2_ configurations. (a) and (b) are schematic diagrams of the structures and path annotations. Red arrows indicate the diffusion direction, and blue and pink balls represent different atomic species, respectively. (c) and (d) are migration barrier curves for Li^+^, Na^+^, and K^+^ along paths 2 and 3. Curves of different colors correspond to different ion species. Barrier values are shown in Table S3; Table S1 shows the Bader charge analysis results for Sc, Si, and N atoms. The table lists the average Bader electron number, the corresponding valence electron number (ZVAL), and the calculated net charge (net charge = valence electron number - Bader electron number) for each atom; Table S2: Binding energies for metal (Li, Na) atoms at the S1, S2, S3, and S4 sites on 2D β_1_-ScSi_2_N_4_ and β_2_-ScSi_2_N_4_; Table S3: Sequential Binding Energies ($E_{sb}$) for Li, Na, and K adsorption on β_1_- and β_2_-ScSi_2_N_4_; Table S4: Migration energy barriers of Li^+^, Na^+^, and K^+^ along different diffusion paths in the β_1_ and β_2_ configurations.
