# Supplementary material for: Diffusion of Alkaline Metals in Two-Dimensional β1-ScSi2N4 and β2-ScSi2N4 Materials: A First-Principles Investigation
Source: Nanomaterials (Basel). 2025 Aug 16;15(16):1268. doi: 10.3390/nano15161268 (PMC12388263; doi:10.3390/nano15161268)
Supplement: Supplementary file 1 [file nanomaterials-15-01268-s001.zip › nanomaterials-3797146-supplementary.pdf]

Supplementary Information

Diffusion of Alkaline Metals in Two-Dimensional β_1-$ScSi_2N_4$ and β_2-$ScSi_2N_4$ Materials: a First Principle Investigation

Index **Page**

1.Favourable adsorption sites of β_1-$ScSi_2N_4$ and β_2-$ScSi_2N_4$ 2

2.Maximum adsorption configurations of Li, Na, and K atoms on β_1-$ScSi_2N_4$ and β_2-$ScSi_2N_4$ monolayers 3

3.Ion diffusion paths and corresponding migration barrier curves forβ_1-$ScSi_2N_4$ and β_2-$ScSi_2N_4$ configurations 4

4. Shows the Bader charge analysis results for Sc, Si, and N atoms of β_1-$ScSi_2N_4$ and β_2-$ScSi_2N_4$ 5

5.Binding energies for metal (Li, Na) atom of β_1-$ScSi_2N_4$ and β_2-$ScSi_2N_4$ 6

6.Sequential Binding Energies (E_{sb}) of β_1-$ScSi_2N_4$ and β_2-$ScSi_2N_4$ 7

7. Migration energy barriers of β_1-$ScSi_2N_4$ and β_2-$ScSi_2N_4$ 8

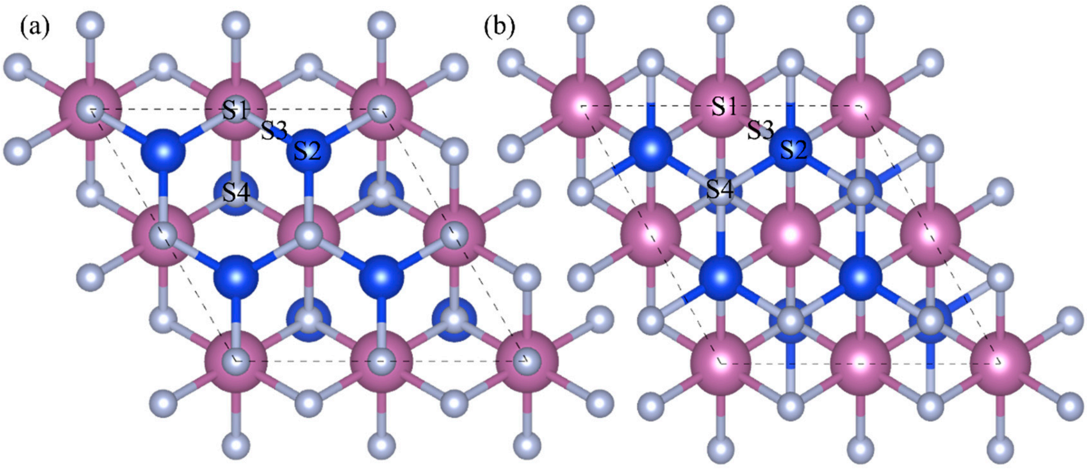

Figure S1. Schematic diagram of the four favourable adsorption sites on the (a)β_1-$ScSi_2N_4$ and (b)β_2-$ScSi_2N_4$. In β_1-$ScSi_2N_4$, the S1, S2, S3, and S4 correspond to the top N atom above the Sc atom, the top of Si, the top of the bridge site between Si and N, the N atom above the Si atom, respectively. In β_2-$ScSi_2N_4$ the S1, S2, S3, and S4 correspond to the top of Sc, the top of Si, the top of the bridge site between Si and Sc, the N atom above the Si atom, respectively.

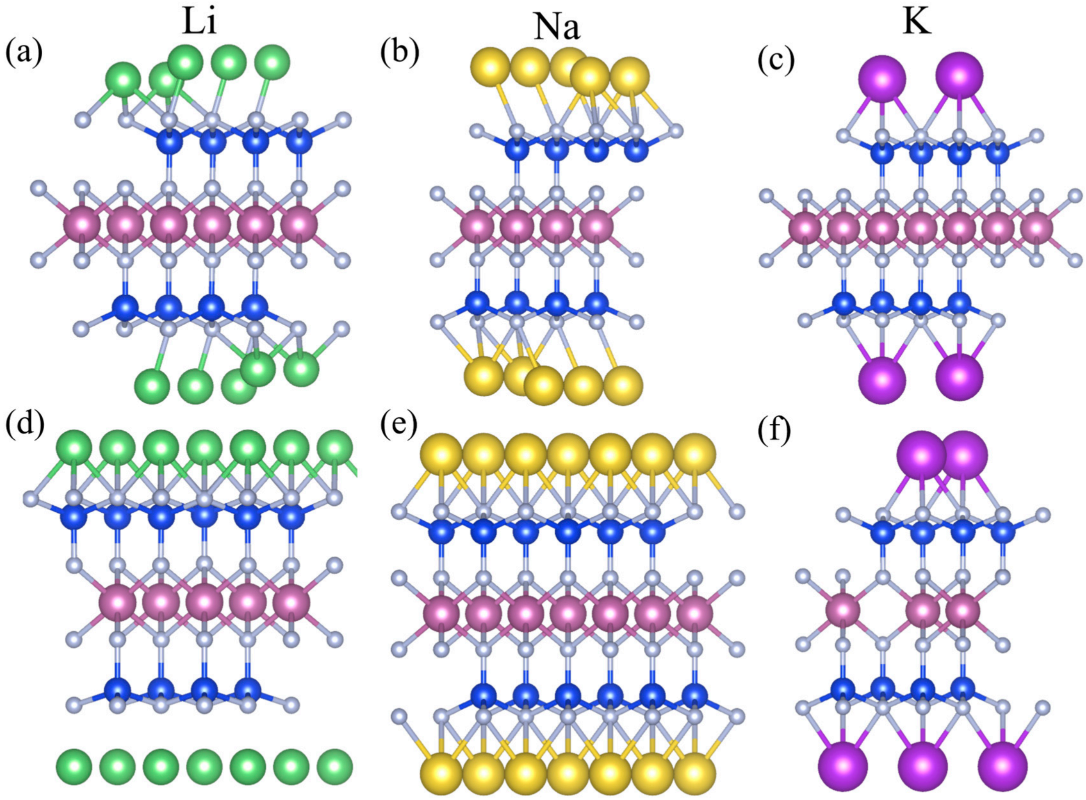

Figure S2. (a-c) Maximum adsorption configurations of Li, Na, and K atoms on β_1-$ScSi_2N_4$ monolayer, and (d-f) on β_2-$ScSi_2N_4$ monolayer, in the order of Li, Na, and K.

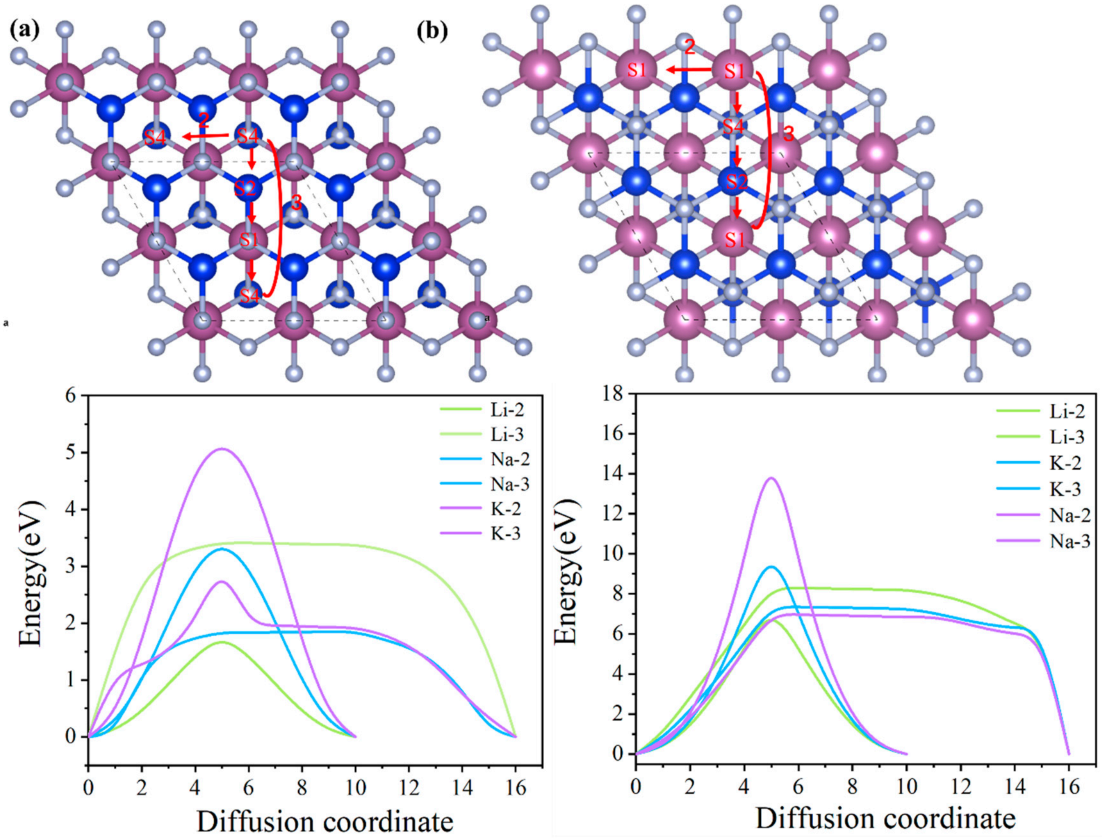

Figure S3. Ion diffusion paths and corresponding migration barrier curves for β_1 and β_2 configurations. (a) and (b) are schematic diagrams of the structures and path annotations. Red arrows indicate the diffusion direction, and blue and pink balls represent different atomic species, respectively. (c) and (d) are migration barrier curves for Li^+, Na^+, and K^+ along paths 2 and 3. Curves of different colors correspond to different ion species. Barrier values are shown in Table S3.

Table S1 Shows the Bader charge analysis results for Sc, Si, and N atoms. The table lists the average Bader electron number, the corresponding valence electron number (ZVAL), and the calculated net charge (net charge = valence electron number - Bader electron number) for each atom.

**Atom Type**	**Average Bader electron number (e)**	**Number of valence electrons (ZVAL)**	**Net charge (e) = ZVAL - Bader electron number**
**β_1-$ScSi_2N_4$**			
Sc	9.19	11	+1.81
Si	6.96	4	-2.96
N	7.95	5	-2.95
**β_2-$ScSi_2N_4$**			
Sc	9.226	11	+1.774
Si	6.901	4	-2.901
N	7.9859	5	-2.9859

Table S2. Binding energies for metal (Li, Na) atom at the S1、S2、S3、S4 sites on 2D β_1-$ScSi_2N_4$ and β_2-$ScSi_2N_4$.

		Li（eV）	Na(eV)
β_1-$ScSi_2N_4$	S1	3.334	2.578
	S2/S3	3.604	2.907
	S4	3.969	3.10
β_2-$ScSi_2N_4$	S1/S3	3.274	3.25
	S2	3.064	3.05
	S4	2.71	2.72

Table S3. Sequential Binding Energies (E_{sb}) for Li, Na, and K Adsorption on β_1- and β_2-$ScSi_2N_4$.

**β_1-$ScSi_2N_4$**					
**Li (x)**	**Esb(x) (eV/Li)**	**Na (x)**	**Esb(x) (eV/Na)**	**K (x)**	**Esb(x) (eV/K)**
1	3.604	1	2.914	1	2.96
2	3.584	2	2.859	2	2.885
4	2.9565	4	2.194	4	1.9925
8	1.34775	8	1.385	8	0.18125
**β_2-$ScSi_2N_4$**					
**Li（x）**	**Esb(x) (eV/Li)**	**Na（x）**	**Esb(x) (eV/Na)**	**K (x)**	**Esb(x) (eV/K)**
1	2.264	1	3.264	1	2.96
2	4.114	2	3.189	2	3.23
4	3.229	4	2.214	4	1.9625
8	2.029	8	1.566	8	0.3775

Table S4. Migration energy barriers of Li^+, Na^+, and K^+ along different diffusion paths in the β_1 and β_2 configurations.

**Configuration**	**ion**	**Path 2 Barrier (eV)**	**Path 3 Barrier (eV)**
β_1	Li^+	1.78	3.43
β_1	Na^+	3.45	1.85
β_1	K^+	5.23	3.09
β_2	Li^+	7.41	8.31
β_2	Na^+	10.60	7.40
β_2	K^+	15.89	7.00
